# PLGA-Nano-Encapsulated Disulfiram Inhibits Cancer Stem Cells and Targets Non-Small Cell Lung Cancer In Vitro and In Vivo

**DOI:** 10.3390/biom14121651

**Published:** 2024-12-23

**Authors:** Kate Butcher, Zhipeng Wang, Sathishkumar Kurusamy, Zaixing Zhang, Mark R. Morris, Mohammad Najlah, Christopher McConville, Vinodh Kannappan, Weiguang Wang

**Affiliations:** 1 Faculty of Science and Engineering, University of Wolverhampton, Wolverhampton WV1 1LY, UK; 2 Disulfican Ltd., Wolverhampton WV9 5HD, UK; 3 School of Biosciences, Division of Natural Sciences, University of Kent, Canterbury CT2 7NZ, UK; 4 Department of Otorhinolaryngology Head and Neck Surgery, Affiliated Hospital of North China University of Science and Technology, Tangshan 063000, China; 5 Faculty of Health, Medicine and Social Care, Anglia Ruskin University, Cambridge CB1 1PT, UK; mohammad.najlah@aru.ac.uk; 6 School of Biomedical Sciences, Ulster University, Coleraine BT52 1SA, UK

**Keywords:** non-small cell lung cancer, cancer stem cells, chemoresistance, disulfiram, copper gluconate, PLGA nano-delivery

## Abstract

Cancer stem cells (CSCs) play a key role in non-small cell lung cancer (NSCLC) chemoresistance and metastasis. In this study, we used two NSCLC cell lines to investigate the regulating effect of hypoxia in the induction and maintenance of CSC traits. Our study demonstrated hypoxia-induced stemness and chemoresistance at levels comparable to those in typical CSC sphere culture. Activation of the NF-κB pathway (by transfection of NF-κB-p65) plays a key role in NSCLC CSCs and chemoresistance. Disulfiram (DS), an anti-alcoholism drug, showed a strong *in vitro* anti-CSC effect. It blocked cancer cell sphere reformation and clonogenicity, synergistically enhanced the cytotoxicity of four anti-NSCLC drugs (doxorubicin, gemcitabine, oxaliplatin and paclitaxel) and reversed hypoxia-induced resistance. The effect of DS on CSCs is copper-dependent. A very short half-life in the bloodstream is the major limitation for the translation of DS into a cancer treatment. Our team previously developed a poly lactic-co-glycolic acid (PLGA) nanoparticle encapsulated DS (DS-PLGA) with a long half-life in the bloodstream. Intra venous injection of DS-PLGA in combination with the oral application of copper gluconate has strong anticancer efficacy in a metastatic NSCLC mouse model. Further study may be able to translate DS-PLGA into cancer applications.

## 1. Introduction

Lung cancer is the second most common malignancy and the leading cause of cancer-related deaths worldwide, with an estimated 2.2 million new cases and 1.8 million deaths. Non-small cell lung cancer (NSCLC) accounts for about 80–85% of all lung cancer cases [1,2]. Despite advances in lung cancer screening, more than 75% of NSCLC patients are diagnosed at a locally advanced or metastatic stage [3]. Although the therapeutic outcomes of NSCLC have been significantly improved in the last two decades, with the development of treatments against driver mutations and immune check point inhibitors, the 5-year survival rate of NSCLC patients remains at 26% in all stages and only 8% in distant metastatic patients [2]. Chemoresistance remains one of the major causes of therapeutic failure in NSCLC. Therefore, elucidation of underlying resistant mechanisms to currently available drugs and the development of novel anti-NSCLC drugs to reverse chemoresistance are of clinical importance and urgency.

Increasing evidence suggests that a small population of cells known as cancer stem cells (CSCs) plays a pivotal role in de novo and acquired resistance to chemo and targeted therapies [4]. NSCLC is a highly heterogeneous tumor with cells expressing putative stem cell markers such as aldehyde dehydrogenase (ALDH), ABCG2, CD44, CD133 and embryonic stem cell (ESC) markers, e.g., Sox2, Oct4 and Nanog [5]. These cells are resistant to all currently available anticancer drugs and are responsible for cancer metastasis, due to their self-renewal ability, uncontrolled proliferation and genomic instability [4]. Therefore, targeting CSCs may improve the therapeutic outcomes of NSCLC patients. Recent studies demonstrate that CSCs are located in hypoxic niches where hypoxic signaling plays a crucial role in the induction and maintenance of their stemness [6]. CSCs exist in a transient and reversible CSC-like state rather than a permanent entity and can originate from the dedifferentiation of cancer cells driven by various factors such as a hypoxic tumor microenvironment [7]. Hypoxia induces epigenetic alterations which change the phenotype of the cancer cells and make them acquire CSC characteristics and chemoresistance [8,9]. Elucidation of the molecular links between hypoxia and CSCs may identify potential targetable CSC-manipulating pathways to overcome chemoresistance in NSCLC. The cellular response to a hypoxic stimulus activates nuclear factor-κB (NF-κB), a principal transcription factor [10]. The NF-κB pathway is activated by numerous external stimuli, oncogenic mutations and various crosstalk signals [11,12] and is constitutively activated in CSCs to promote various survival mechanisms [13,14,15]. Suppression of the NF-κB pathway decreases CSC characteristics, sensitizes cancer cells to chemotherapy, inhibits tumor growth and blocks metastasis [8,16,17,18].

Therefore, the development of drugs that target the CSC population is key to improving the therapeutic outcomes for NSCLC patients. New drug discovery and development is a time-consuming and expensive procedure. Anticancer drug development has a persistent attrition rate of 95% due to safety issues [19]. Drug repurposing is an ideal process to identify new uses for FDA-approved drugs outside of the original intended medical use [20]. Disulfiram (DS) is an anti-alcoholism drug used in the clinic for more than 70 years with excellent safety records [21]. DS has been demonstrated significant anticancer activity in various cancers *in vitro* and *in vivo* [18,22,23,24,25]. Previous studies revealed that the cytotoxicity of DS is entirely copper (Cu) dependent [26,27]. The chelation of DS with Cu generates reactive oxygen species (ROS) which are highly toxic to cancer cells. Both DS plus Cu and copper-diethyldithiocarbamate (Cu-DDC), the final product of the Cu and DS chelation, inhibit NF-κB activity and target CSCs [16,17,22,23]. The sulfhydryl group of DS is essential for its reaction with Cu and anticancer activity [26]. Only an oral version of DS is currently available in the clinic [21]. After oral administration, DS is enriched and metabolized in the liver where the sulfhydryl group of DS is promptly methylated, glucuronidated and degraded and loses its anticancer efficacy in cancer patients [26]. This explains the discrepancy between the anticancer activities of DS in the laboratory and in cancer clinical trials. Therefore, the very short half-life of DS in the bloodstream became the bottleneck for the translation of DS into cancer treatment. In order to apply DS to cancer treatment, we first suggested using the nano-drug-delivery system to protect the sulfhydryl group and deliver the intact DS into cancer tissues [28]. We have previously shown that encapsulation of DS into liposome or poly lactic-co-glycolic acid (PLGA) significantly extends the half-life of DS and improves its *in vivo* anticancer efficacy in mouse breast, liver cancer and glioblastoma models [8,18,23].

In this study, we demonstrate that hypoxia-induced CSCs are responsible for chemoresistance in NSCLC cell lines. NF-κB plays pivotal roles in hypoxia-induced cancer cell stemness and chemoresistance. DS/Cu strongly targets CSCs and demonstrated high anti-NSCLC activity *in vitro*. In combination with copper, PLGA encapsulated DS (DS-PLGA) showed very strong *in vivo* anti-NSCLC efficacy in a mouse metastatic model. Our data indicated that further study may translate DS-PLGA into NSCLC clinical treatment.

## 2. Materials and Methods

### 2.1. Cell Lines and Reagents

The NSCLC cell lines A549 and NCI-H23 were purchased from ATCC (Teddington, UK). DS, copper (II) chloride (CuCl_2_), copper gluconate (CuGlu), doxorubicin (DOX), gemcitabine (dFdC), oxaliplatin (OXA), paclitaxel (PTX), poly-2-hydroxyethyl methacrylate (poly-HEMA), propidium iodide (PI) were purchased from Sigma (Dorset, UK). DS-PLGA was developed and characterized in our lab [23].

### 2.2. 1-(4,5-. Dimethylthiazol-2-yl)-3,5-Diphenylformazan (MTT) Cytotoxicity Assay and CI-Isobologram Analysis

All cell lines were cultured in DMEM (Lonza, Wokingham, UK) supplemented with FBS (10%), L-glutamine (2 mM), penicillin/streptomycin/amphotericin (1%, *v*/*v*) (Lonza, Slough, UK) and maintained in normoxic conditions at 37 °C and 5% CO_2_. Hypoxic-cultured cells were incubated at 37 °C, 5% CO_2_ and 1% O_2_ for 4 days. For cytotoxicity assay, cells (2.5 × 10^4^ cells/mL) were cultured in 96-well flat-bottomed microtiter plates overnight and then exposed to drugs for 72 h in both normoxic and hypoxic conditions before subjected to a standard MTT assay [29]. CI isobologram was used to determine the synergistic effect between anticancer drugs and DS/Cu and calculation of Combination Index (CI). Cells were exposed to various concentrations of anticancer drugs, DS/Cu or in a combination of anticancer drug and DS/Cu [consistent concentration (10 μM) of CuGlu] and then subjected to MTT assay. A constant ratio of anticancer drug:DS of 1:1 (DOX, dFdC and PTX) or 100:1 (OXA) was used. The combined cytotoxicity was determined by CI isobologram using CalcuSyn software (Biosoft, Cambridge, UK) [30]. Mutually exclusive equations were used to determine the CI.

All cell lines were cultured in DMEM (Lonza, Wokingham, UK) supplemented with FBS (10%), L-glutamine (2 mM), penicillin/streptomycin/amphotericin (1%, *v*/*v*) (Lonza, Slough, UK) and maintained in normoxic conditions at 37 °C and 5% CO_2_. Hypoxic-cultured cells were incubated at 37 °C, 5% CO_2_ and 1% O_2_ for 4 days. For cytotoxicity assay, cells (2.5 × 10^4^ cells/mL) were cultured in 96-well flat-bottomed microtiter plates overnight and then exposed to drugs for 72 h in both normoxic and hypoxic conditions before subjected to a standard MTT assay [29]. CI isobologram was used to determine the synergistic effect between anticancer drugs and DS/Cu and calculation of Combination Index (CI). Cells were exposed to various concentrations of anticancer drugs, DS/Cu or in a combination of anticancer drug and DS/Cu [consistent concentration (10 μM) of CuGlu] and then subjected to MTT assay. A constant ratio of anticancer drug:DS of 1:1 (DOX, dFdC and PTX) or 100:1 (OXA) was used. The combined cytotoxicity was determined by CI isobologram using CalcuSyn software (Biosoft, Cambridge, UK) [30]. Mutually exclusive equations were used to determine the CI.

### 2.3. Spheroid Culture and Sphere Reformation Assay

The NSCLC cells were cultured as spheres in poly-HEMA-coated ultra-low adherence flasks containing stem cell medium (SCM) which contains DMEM-F12 supplemented with 2 mM L-glutamine, 1% (*v*/*v*) penicillin/streptomycin/amphotericin mix (Lonza), B-27 (Invitrogen, Paisley, UK), 10 ng/mL basic fibroblast growth factor (b-FGF) (R&D system, Abingdon, UK), 10 ng/mL epidermal growth factor (EGF, Sigma), 20 μg/mL insulin, 20 μg/mL heparin, and 4.5 g/L D-glucose (Sigma). For the sphere reformation assay, the 6-day-cultured spheroid cells were trypsinised and the single cell suspension was exposed to different drugs for 6 h at 37 °C, 5% CO_2_. The treated cells were cultured in drug-free medium at a low cell density (2000 cells/well) in poly-HEMA-coated 24 well plates for 7 days. The total numbers of the reformed spheres were counted and images were taken at 4× magnification.

### 2.4. Clonogenic Assay

Normoxic and hypoxic-cultured cells were exposed to IC_50_ concentrations of DOX, dFdC, OXA, PTX, Cu, DS and DS/Cu for 48 h. The drug-treated cells (5 × 10^3^/well) were re-seeded in 6-well plates and cultured in drug-free medium under normoxic and hypoxic conditions, respectively, for 10 days. The cells were fixed and stained with crystal violet. The colonies containing at least 50 cells were counted.

### 2.5. Detection of CSC Markers

The activity and expression levels of ALDH, CD133 and ABCG2 were determined in spheroid, normoxia and hypoxia-cultured A549 and NCI-H23 cells. The CSC markers in spheroid and hypoxia-cultured cells were also examined after 6 h DS/Cu treatment. The ALDH-positive population was detected by the ALDEFLUOR kit (StemCell Tech, Cambridge, UK) following the supplier’s instruction. The specificity of the kit was verified with diethylaminobenzaldehyde (DEAB) treatment. The CD133 and ABCG2-positive populations were detected by FITC conjugated anti-CD133 (Macs Miltenyi Biotec, Surrey, UK) and APC conjugated anti-ABCG2 (BD Biosciences, Berkshire, UK) antibodies.

### 2.6. Measurement of the Hypoxic Cell Population

The Hypoxyprobe^TM^-1 Plus Kit (Hypoxyprobe, Inc, Massachusetts, USA) was used to detect and quantify the hypoxic population in A549 and NCI-H23 cell lines. The cells were incubated overnight in a 100 μM HPP-containing medium. The cells were fixed with ice-cold ethanol and stained with anti-HPP-FITC antibody (1:1000) in flow buffer in the dark and then analyzed by flow cytometry. For immunocytochemistry assay, the cells were fixed using an Image-iT^®^ Fix Perm Kit (Invitrogen, Waltham, MA, USA) and stained with anti-HPP-FITC antibody (1:100) and counterstained with PI.

### 2.7. Western Blot Analysis

The expression levels of NF-κB-p65 in whole and nuclear protein were determined using primary NF-κB-p65 antibody (Abcam, Cambridge, UK) and HRP conjugated secondary antibody (Sigma). β-actin (Sigma) (1:5000) and nucleolin (Santa Cruz, TX, USA) (1:1000) were used to detect housekeeping proteins in whole and nuclear proteins, respectively. The Western blotting signal was detected using the EZ-ECL enhanced chemiluminescence kit (Biological Industries, Cromwell, CT, USA).

### 2.8. Quantitative Real Time RT-PCR (qRT-PCR)

qRT-PCR was performed using Taqman™ assays according to the manufacturer’s instructions. RNA was extracted from cultured NSCLC cells using an RNA purification kit (Norgen Biotek, Thorold, ON, Canada)) following the manufacturer’s instructions, as a template for cDNA synthesis using Multiscribe Reverse Transcriptase (Applied Biosystems, Woburn, MA, USA). The following primers/probes were used: Taqman™ gene expression assay ID P65 Hs01042014_m1, HIF1A Hs00936371_m1, HIF2A Hs01026149_m1, SOX2 Hs01053049_s1, OCT4 Hs00999632_g1, NANOG Hs02387400_g1, HPRT1 Hs99999909_m1. Gene expression was normalized for control gene expression (HPRT1) and calculated according to the comparative 2^−^^ΔΔCT^ method.

### 2.9. Luciferase Reporter Gene Assay

A dual luciferase assay kit was used for luciferase reporter gene assay in 96-well plates following the instruction from the supplier (Promega, Southampton, UK). The transcriptional activity of NF-κB and hypoxia response element (HRE) was determined using pNFκB-Tal-Luc (BD Biosciences) and pGL4.4-LUC2p/HRE (Promega) luciferase vectors, respectively. pGL3-Basic (BD Biosciences) vector was used as the background control. All of the luciferase vectors were co-transfected with pSV40-Renilla (BD Biosciences) an internal control for normalization of the transcriptional activity. The luciferase activity in each well was normalized to pSV40-Renilla using Ln = L/R (Ln: normalized luciferase unit; L: luciferase activity reading; R: Renilla activity reading). The transcriptional specificity was monitored by the transcriptional activity of the pGL3-Basic. All transfections were performed in triplicate with at least duplicate independent experiments.

### 2.10. NSCLC Xenograft and In Vivo DS-PLGA Treatment

Five-week-old female BALB/c Nu/Nu athymic nude mice (Biotechnology and Cell Biology Shanghai, Shanghai, China) were housed under pathogen-free conditions according to the animal care guidelines of Fourth Military Medical University (FMMU) China. All experiments were reviewed and approved by the FMMU Ethical Committee. The A549 NSCLC metastatic xenograft model was used in this study. A549 cells (5 × 10^6^ in 100 μL of PBS) were injected into the tail vein. Three days after tumor cells injection, the tumor-bearing mice were randomly subdivided into 2 groups (7 mice/group) and subjected to the following treatments. 1. Control: no treatment; 2. CuGlu 5 mg/kg p.o and DS-PLGA 5 mg/kg i.v (4 h after CuGlu) 2 times per week for 3 weeks. The mouse body weight was monitored three times per week and all the mice were sacrificed on day 21. The lungs were dissected, weighted and then formalin-fixed for paraffin embedment, sectioning and H&E staining.

### 2.11. Statistical Analysis

The software GraphPad Prism was used for statistical analysis. The statistical significance of treatment outcomes was assessed using the Student’s *t*-test and one-way ANOVA; *p*-value scores of <0.05 (*) and <0.01 (**) were considered statistically significant. Data are displayed as mean values ± standard deviation.

## 3. Results

### 3.1. Spheroid-Cultured NSCLC Cells Are Hypoxic with CSC Characters and Resistant to Anti-NSCLC Drugs

**Figure 1:**
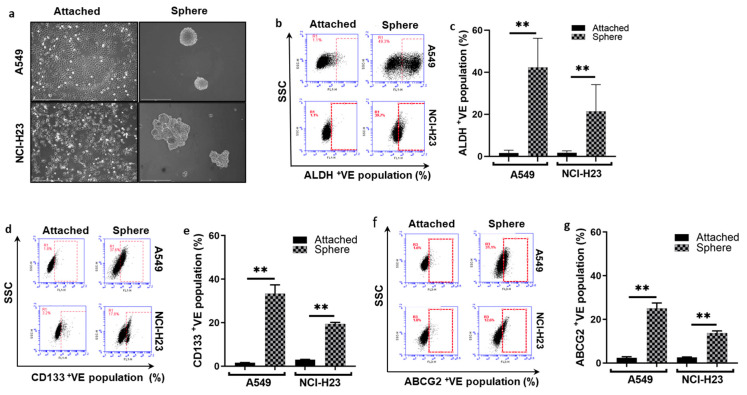

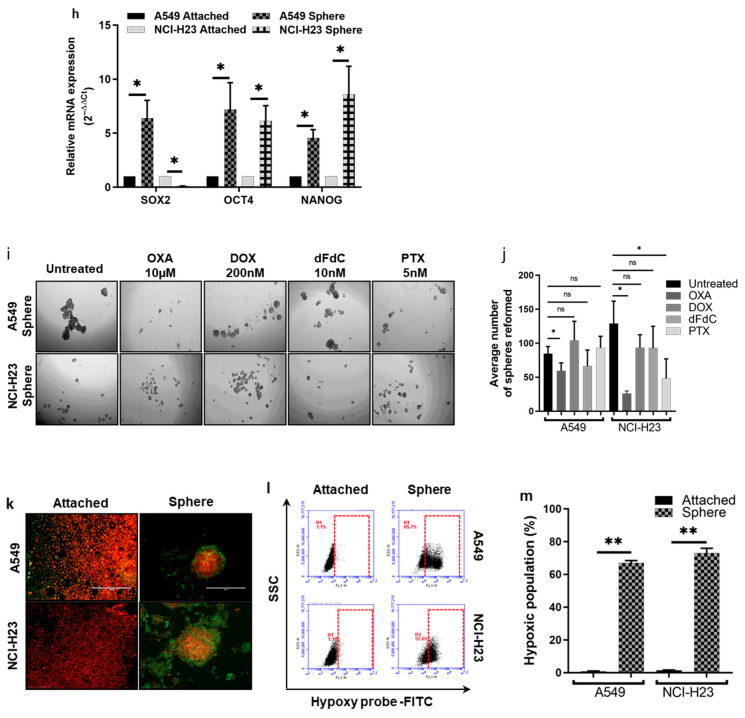
Sphere-cultured NSCLC cells demonstrate CSC and ESC markers, chemoresistance and contain high proportion of hypoxic cell population. (**a**) Morphology of the attached and sphere-cultured cells (×10 magnification). (**b**–**g**). Flow cytometric analysis of the expression of CD133, ALDH and ABCG2 CSC markers in attached and sphere-cultured NSCLC cells (mean ± SD; *n* = 3). (**h**) qRT-PCR analysis of ESC markers in attached and sphere-cultured cells (mean ± SD; *n* = 3). (**i**,**j**) Effect of four chemotherapy drugs on the sphere reformation ability (×10 magnification, mean ± SD; *n* = 3). (**k**) Morphology of hypoxic cells in the spheres (×10 magnification). Hypoxic cells were detected by Hypoxyprobe and FITC-conjugated anti-Hypoxyprobe MAb staining at Exc 492 nm and Emi 520 nm (green, cytoplasm). The nuclei were counterstained by PI (red) Exc 530 nm and Emi 618 nm. (**l**,**m**) Flow cytometry comparison of the proportions of Hypoxyprobe stained hypoxic population in monolayer and sphere-cultured cells (mean ± SD; *n* = 3). *ns = non-significant;* * *p* < 0.05 and ** *p* < 0.01.

In this study, we used spheroid cultures to induce the CSC population in two NSCLC cell lines. After 6 days of culture in SCM at low attachment condition, A549 and NCI-H23 cells formed a distinct spheroid morphology (Figure 1a). In comparison with the attached cells, a significantly larger population of the sphere-cultured cells expresses ALDH^+^, CD133^+^ and ABCG2^+^ (Figure 1b–g). The stemness of the sphere-cultured cells was further proved by the detection of other ESC markers, e.g., SOX2, OCT4 and NANOG, using RT-PCR (Figure 1h). Furthermore, the sensitivity of the spheroid-cultured cells to the four most commonly used NSCLC chemotherapy drugs was examined using the sphere reformation assay (Figure 1i,j). DOX and dFdC did not inhibit the sphere reformation in both cell lines and no inhibition by PTX was observed in the A549 cell line. Although the sphere reformation was inhibited by OXA in both cell lines and PTX in the NCI-H23 cell line, significant numbers of the reformed spheres were still detected. These results demonstrate that sphere-cultured CSCs are resistant to anti-NSCLC drugs. The hypoxic status in the sphere-cultured cells was examined using Hypoxyprobe immunocytochemistry staining and flow cytometry analysis. In comparison to the attached cells, a significantly higher proportion of hypoxic cells were detected in the sphere-cultured cells (Figure 1k–m).

### 3.2. Hypoxia-Cultured NSCLC Cells Show CSC Traits and Are Resistant to Anticancer Drugs

**Figure 2:**
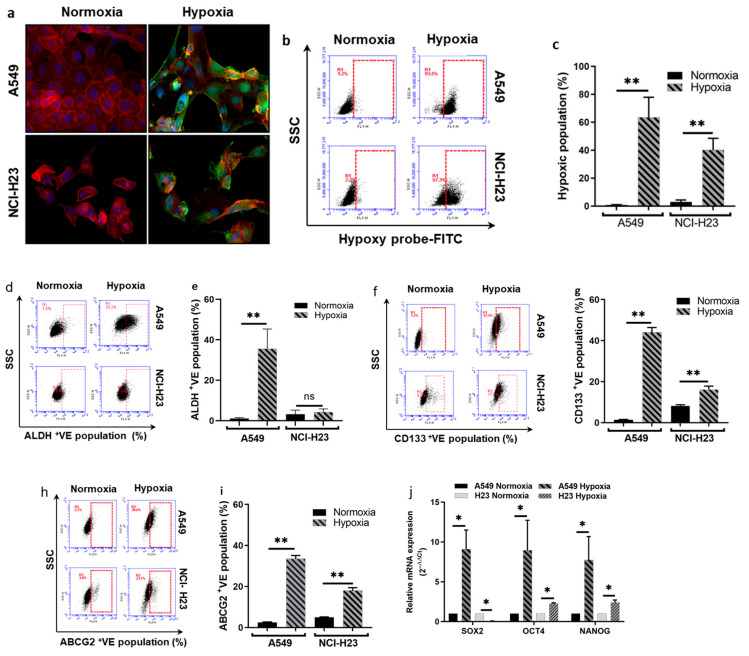

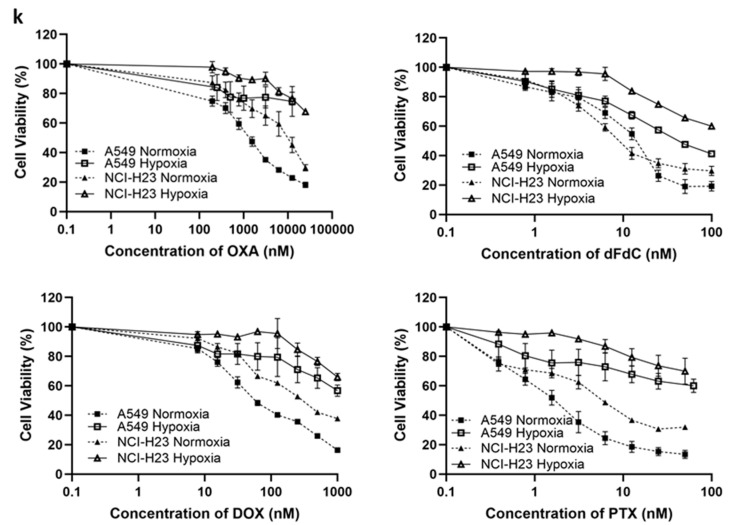
Hypoxic culture induces expression of CSC and ESC markers and chemoresistance. (**a**) Images of Hypoxyprobe-stained monolayer cells cultured in normoxic and hypoxic conditions (×40 magnification). (**a**) Hypoxic cells were detected by Hypoxyprobe and FITC-conjugated anti-Hypoxyprobe MAb staining (Exc 492 nm and Emi 520 nm; green, cytoplasm). The nuclei were counterstained by DAPI (Exc 345 nm and Emi 455 nm; Blue). The cytoplasm was stained with ActinRed 555 (Exc 540 nm and Emi 565 nm; red, cytoplasm) (**b**,**c**) Flow cytometric analysis of Hypoxyprobe-stained cells (mean ± SD; *n* = 3). (**d**–**i**) Flow cytometric analysis of the expression of CD133, ALDH and ABCG2 CSC markers in attached and sphere-cultured cells (mean ± SD; *n* = 3). (**j**) qRT-PCR analysis of ESC markers in attached and sphere-cultured cells (mean ± SD; *n* = 3). (**k**) Comparison of cytotoxicity of four chemotherapy drugs in normoxia and hypoxia-cultured cells (mean ± SD; *n* = 3). *ns = non-significant;* * *p* < 0.05, ** *p* < 0.01.

Hypoxia and CSC traits were co-detected in sphere-cultured cells (Figure 1). To determine the relationship between hypoxia and CSC traits, we grew the monolayer-cultured cancer cell lines in hypoxic conditions. The hypoxia was confirmed using the Hypoxyprobe kit. Figure 2a shows the immunocytochemistry image of the hypoxia-cultured monolayer cells. Flow cytometric analysis demonstrated a significantly higher percentage of hypoxic cells after being cultured in hypoxia conditions (Figure 2b,c). In comparison with normoxia-cultured cells, a significantly higher percentage of CSC markers (ALDH, CD133 and ABCG2) positive cells was detected in hypoxia-cultured cells (Figure 2d–i). Significantly higher levels of mRNA for ESC markers (SOX2, OCT4 and NANOG) were detected in hypoxia-cultured cells (Figure 2j). The cytotoxicity of DOX, dFdC, OXA and PTX after 72 h treatment was analysed using the MTT assay (Figure 2k). The IC_50_ values of the anti-NSCLC drugs in normoxia and hypoxia-cultured cancer cell lines are shown in Table 1. Hypoxia-cultured cells are highly resistant to all of these clinically used drugs. These results suggest that hypoxia-cultured NSCLC cells developed more chemoresistant CSCs.

The normoxia and hypoxia-cultured cells were exposed to different drugs for 72 h and subjected to MTT assay.

### 3.3. NF-κB Pathway Plays a Pivotal Role in CSC Traits and Chemoresistance

**Figure 3:**
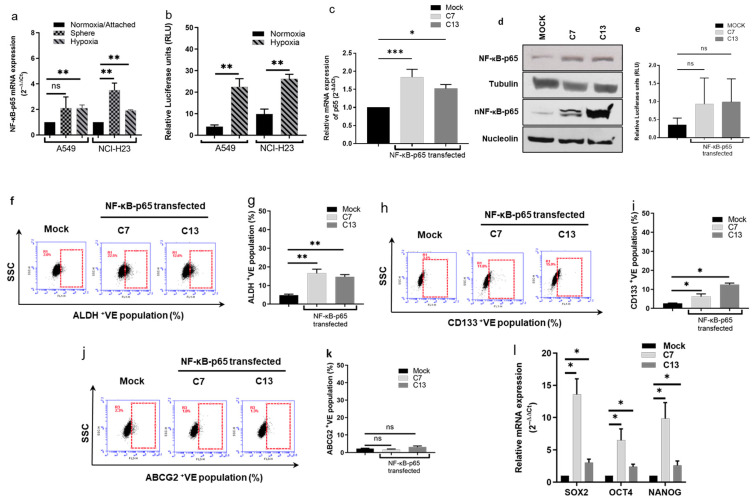

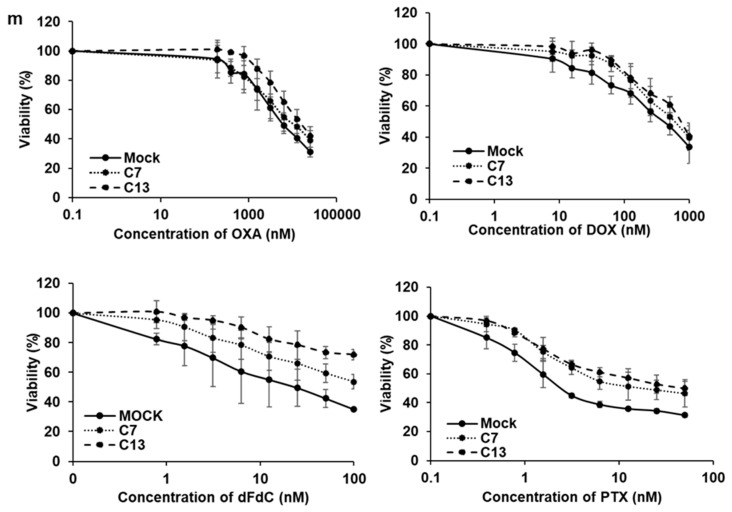
Stable transfection of NFκB-p65 induces the expression of CSC markers and chemoresistance in the A549 cell line. (**a**,**b**) Hypoxic culture induces NFκB-p65 mRNA expression and NFκB transcriptional activity. (**c**–**e**) qRT-PCR and Western blot show that NFκB-p65 stably transfected clones express high levels of NFκB-p65 mRNA (**c**) and protein (**d**) and demonstrate high NFκB transcriptional activity (**e**). (**f**–**k**) Flow cytometry detection of the expression status of CSC markers in the NFκB-p65 transfected clones. (**l**) qRT-PCR detection of the mRNA expression of the ESC markers in NFκB-p65 transfected clones. (**m**) MTT analysis of the cytotoxicity of four chemotherapy drugs on the NFκB-p65 transfected clones (mean ± SD; *n* = 3). *ns = non-significant;* * *p* < 0.05, ** *p* < 0.01. Western blot original images are in the supplementary materials.

In comparison with normoxia-cultured cells, both hypoxia and sphere-cultured A549 and NCI-H23 cell lines express significantly higher levels of NF-κB-p65 mRNA, the key player of the canonical NF-κB activation pathway involving in chemoresistance (Figure 3a). The luciferase reporter gene assay demonstrated significantly higher NF-κB transcriptional activity in hypoxia-cultured cell lines (Figure 3b). These results are in line with our previous findings in human glioblastoma cell lines [8] and indicate the relationship between NF-κB activation and hypoxia-induced NSCLC CSCs. To investigate the causal relationship between the NF-κB pathway, CSC traits and chemoresistance, we stably transfected pcDNA3.1/NF-κB-p65 plasmid into the A549 cell line to overexpress NF-κB-p65. The pcDNA3.1 empty vector-transfected cells were used as mock control. Compared with a mock clone, two positive clones (C7 and C13) expressed significantly higher NF-κB-p65 mRNA and protein (whole and nuclear) (Figure 3c,d). Higher NF-κB activity was also detected by luciferase reporter gene assay (Figure 3e). In comparison with the mock clone, the ALDH activity and CD133 expression were significantly induced by NF-κB-p65 transfection in C7 and C13 clones (Figure 3f–i). There was no significant difference in the expression of ABCG2 (Figure 3j,k). The RT-PCR results also show an increase in the mRNA levels of the ESC transcription factors SOX2, OCT4 and NANOG in the NF-κB-p65 transfected clones (Figure 3l). These results strongly indicate the positive causal relationship between NF-κB activity and CSC traits in NSCLC cells. Chemoresistance is one of the major characteristics of hypoxia-induced CSCs. To establish the role of NF-κB in chemoresistance, the cytotoxicity of anti-NSCLC drugs in NF-κB-p65 transfected A549 cell lines was compared with that in the mock clone. The NF-κB-p65 transfected clones demonstrate resistance to dFdC, OXA, PTX and DOX, although it does not reach statistical significance in DOX-treated cells (Figure 3m and Table 2).

### 3.4. DS/Cu Targets CSCs and Reverses Chemoresistance in NSCLC Cell Lines

**Figure 4:**
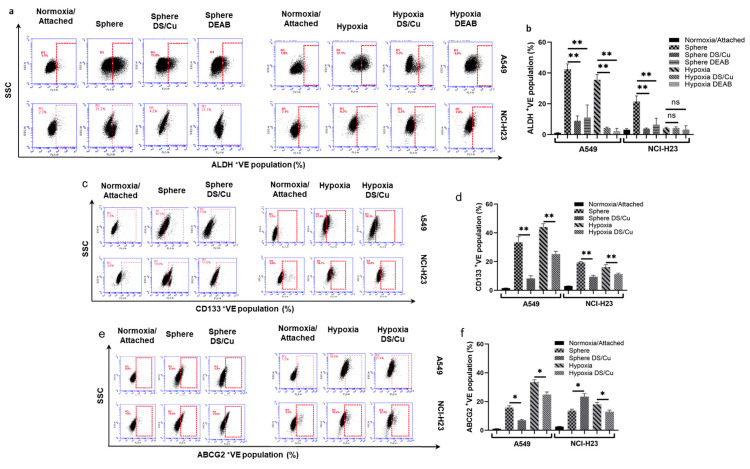

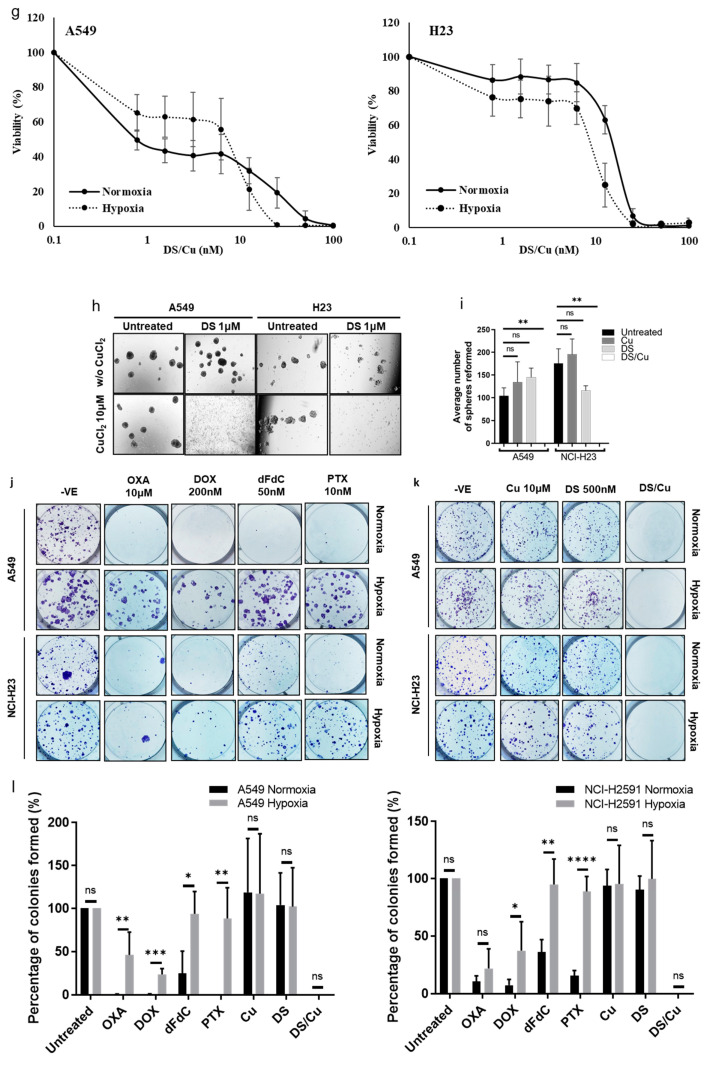
DS/Cu targets the CSC population and reverses chemoresistance in vitro. (**a**–**f**) DS/Cu inhibits the expression of CSC markers in sphere and hypoxia-cultured cells (mean ± SD; *n* = 3). (**g**) MTT analysis of DS/Cu on normoxia and hypoxia-cultured cell lines. (**h**,**i**) DS/Cu blocks sphere reformation ability in NSCLC cell lines (mean ± SD; *n* = 3). (**j**–**l**) Clonogenic assay: The chemotherapy drugs inhibit clonogenicity in normoxia-cultured cells but not in hypoxia-cultured cells (**j**). The clonogenicity in both cell lines was blocked by DS/Cu in both normoxic and hypoxic conditions in a copper-dependent manner (**k**). The colony numbers were counted and analyzed (mean ± SD; *n* = 3) (**l**). *ns = non-significant;* * *p* < 0.05; ** *p* < 0.01; *** *p* < 0.001; **** *p* < 0.0001.

The above data strongly suggest that NF-κB plays a critical role in maintaining CSC status and chemoresistance. Our previous findings indicate that DS/Cu inhibits NF-κB activity, targets CSCs and reverses chemoresistance in other types of cancer [8,18,27]. Here, we examine the cytotoxic effect of DS/Cu on CSCs in A549 and NCI-H23 cell lines. Most of the sphere- and hypoxia-induced CSC markers in A549 cell lines are significantly inhibited by DS/Cu. DS/Cu inhibits the expression of ALDH, CD133 and ABCG2 in the sphere and hypoxia-cultured A549 cells (Figure 4a–f). The expression of ALDH and CD133 in sphere-cultured NCI-H23 cells is inhibited by DS/Cu (Figure 4a–d). The expression of CD133 and ABCG2 in hypoxia-cultured NCI-H23 cells is also inhibited by DS/Cu (Figure 4c–f). Unexpectedly, the expression of ALDH in NCI-H23 cells was not induced by hypoxic culture (Figure 2b) and DS/Cu did not show an inhibiting effect on ABCG2 expression in sphere-cultured NCI-H23 cells (Figure 4e,f). Although hypoxia induces resistance to four anti-NSCLC drugs, the cytotoxicity of DS/Cu is comparable in normoxia and hypoxia-cultured cells (Figure 4g). The effect of DS/Cu on sphere reforming ability was examined. DS and Cu, in combination, completely blocked the sphere reformation ability in A549 and NCI-H23 cell lines. In contrast, these effects were not observed in DS or Cu singly treated cells (Figure 4h,i). The clonogenicity is also the character of CSCs. A clonogenic assay was used to determine the clonogenicity of normoxic and hypoxic NSCLC cells treated with NSCLC chemotherapy drugs. The clinically used NSCLC chemotherapy drugs inhibited the clonogenicity in normoxia-cultured cells but the hypoxia-cultured cells were resistant to the treatment (Figure 4j,l). The combination of DS/Cu completely blocked the colony formation in both normoxia and hypoxia-cultured cells. Whereas, the inhibiting effect was not observed in DS or Cu singly treated cells (Figure 4k,l). These results indicate that DS/Cu targets NSCLC CSCs in which Cu is indispensable. Furthermore, the effect of DS/Cu on the hypoxia-induced anti-NSCLC drug resistance in A549 and NCI-H23 cell lines was examined. DS/Cu significantly enhances the cytotoxicity of four first-line anti-NSCLC drugs and completely reverses the hypoxia-induced chemoresistance in both cell lines (Figure 4m and Table 3). Isobologram analysis shows a synergistic combination effect between DS/Cu and anticancer drugs at a wide concentration range (Table 3).

### 3.5. DS-PLGA/Cu Targets Cancer Xenografts in a Metastatic NSCLC Mouse Model

**Figure 5:**
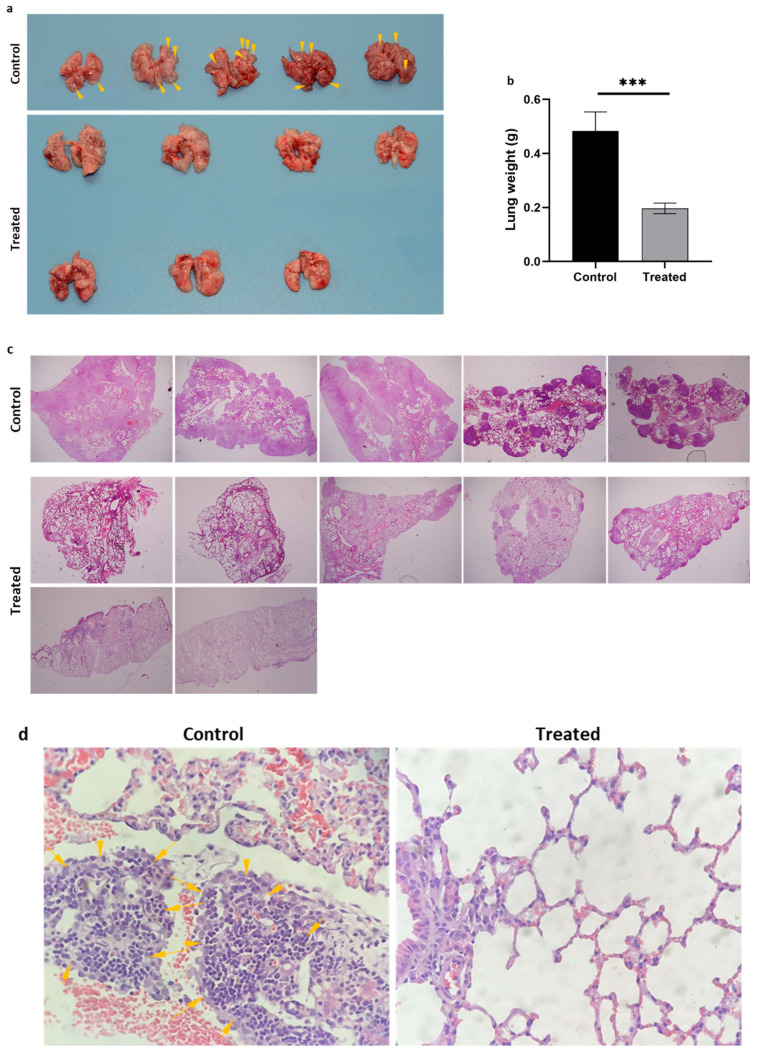
Anti-NSCLC efficacy of DS-PLGA/Cu in mouse NSCLC metastatic xenograft model. (**a**) The macro-morphology of the lungs from control and treated mice. Yellow arrows show tumor nodules. (**b**) The weight of the lungs from control and treated groups. (**c**) The micro-morphology shows multiple tumor nodules in the lung sections of the control mice. No or very few nodules were detected in the sections of the lungs from the treated group (×4 magnification). (**d**) Typical pathological images of the H&E stained lung sections showing cancer cell clusters (yellow arrows) in the lungs of control mice (×100 magnification). *** *p* < 0.001.

To develop an injectable DS, we invented a PLGA-encapsulated DS (DS-PLGA) [23]. Our previous studies show that DS-PLGA significantly improved the half-life of DS in serum [23]. In combination with oral CuGlu, intravenous administration of DS-PLGA demonstrated significant anticancer efficacy in liver cancer and glioblastoma mouse models [8,23]. In this study, the same DS-PLGA formulation was used to assess the anti-NSCLC efficacy in a metastatic A549 NSCLC mouse model. Two mice in the control group (*n* = 7) died on days 15 and 18. All the mice in the treated group (*n* = 7) tolerated the treatment very well and survived to the end of the experiment (day 21). No body weight loss was observed in DS-PLGA/CuGlu-treated mice. The lung macro morphologies in both groups are shown in Figure 5a. Tumor nodules were identified in the lung of the control mice, especially around the edge (arrows in Figure 5a). Due to the cancer cell infiltration, the average lung weight in the control mice is significantly heavier than that in the treated mice (Figure 5b). In comparison with the treated mice, massive cancer cell infiltration (arrows) was observed in the lung, especially around the edge, of the control mice (Figure 5c). Figure 5d shows the typical histological images of the lung in the control and treated groups. Cancer cells were observed in the lung of the control group (arrows).

## 4. Discussion

In combination with immunotherapy and targeted therapy, cytotoxic chemotherapy is currently still the first-line therapeutic arm for advanced/metastatic NSCLC patients [31]. Neoadjuvant or adjuvant chemotherapy often achieves good initial therapeutic outcomes. Due to the acquired chemoresistance, most NSCLC patients will relapse [32]. NSCLC is highly heterogenous containing a very small population of CSCs with increased capacity for invasion, metastasis and chemoresistance [8,17,18,33,34,35]. The first identification of human lung CSCs was reported 40 years ago [36]. Due to the genotypic and histological varieties of lung cancer, lung CSCs have been studied much less than in other types of cancer [34]. In this study, we investigated the chemosensitivity of NSCLC CSCs to the four most used chemotherapeutic agents (PTX, DOX, dFdC and OXA). A classical *in vitro* spheroid suspension CSC culture from two NSCLC cell lines was examined (Figure 1a). In comparison with the attached culture counterparts, the sphere-cultured cells express high levels of CSC markers (CD133, ALDH and ABCG2) (Figure 1b–g). Significantly higher mRNA expression of the embryonic stem cell markers (SOX2, OCT4 and NANOG) was also detected in the sphere-cultured cells (Figure 1h). These results are in line with previous reports [37,38,39,40] indicating that NSCLC CSCs were enriched by spheroid culture. Furthermore, the anti-CSC activity of the four anti-NSCLC chemotherapy drugs was examined using a sphere reformation assay (Figure 1i,j). No inhibition of sphere reformation was observed in both cell lines after treatment with DOX and dFdC as well as the PTX-treated A549 cell line. Although the sphere number was significantly reduced after OXA treatment in both cell lines and PTX in the NCI-H23 cell line, a considerable number of spheres was still reformed in all treated cells. This result is consistent with our previous data derived from other types of cancers [8,16,17,18,22,23] indicating that the chemoresistant NSCLC CSC population is the source of cancer recurrence.

It is widely accepted that both CSCs and somatic stem cells are harboured in a specific microenvironment named stem cell niches [41]. Unlike somatic stem cells of which the differentiation is irreversible, CSC is a dynamically reversible state rather than an entity [8,42]. The molecular and cellular characters of CSCs are modified by microenvironmental factors and the lack of blood vasculature in rapidly expanded solid cancer mass results in the formation of a hypoxic CSC niche [43] which is identified as a hallmark of the tumor microenvironment (TME) [44]. The hypoxic niche plays a pivotal role in the induction and maintenance of the status of CSCs [44]. In line with our previous studies on other types of cancer [8,18], sphere-cultured NSCLC cells showed a hypoxic core region with a high population of hypoxic cells detected (Figure 1k–m). This finding indicates that hypoxia may also play a key role in the maintenance of stemness in NSCLC CSCs. Hypoxia-monolayer-cultured cells expressed high levels of CSC markers and embryonic stem cell markers (Figure 2d–j). In contrast, ALDH and SOX2 were not induced by hypoxic culture. This further indicates that no single marker is completely reliable for NSCLC CSC detection [37]. Consistent with sphere reformation assay results, the hypoxia-cultured cells demonstrated strong resistance to all four chemotherapy drugs (Table 1 and Figure 2k) and showed significantly higher invasive activity (Figure 4i,k).

The activation of the NF-κB pathway plays a central role in the development, survival and proliferation of CSCs in many types of cancers [8,16,18,22,23]. Figure 3a and b show that both sphere and hypoxia-cultured NSCLC cell lines expressed high levels of NF-κB-p65 mRNA. To examine the effect of the NF-κB pathway on NSCLC CSC traits, A549 cells were stably transfected with NF-κB-p65. The transfected clones showed high expression of NF-κB-p65 mRNA, protein expression and NF-κB activity (Figure 3c–e). These clones expressed high levels of ALDH, CD133 and embryonic stem cell markers (Figure 3f–l). The transfected clones are also significantly resistant to all four tested chemotherapy drugs (Table 3 and Figure 3m). These results indicate that the NF-κB pathway plays a key role in the maintenance of stemness in NSCLC CSCs.

DS is a medicine that has been used in alcoholism treatment for over 70 years [45], and it shows very strong anticancer potential [46,47]. Our previous studies indicate that it is a strong NF-κB pathway inhibitor and anti-CSC agent in multiple types of cancer [8,16,17,18,22,23,27,48,49]. In this study, we demonstrated that DS reverses hypoxia-induced stemness in NSCLC cell lines (Figure 4a–f). DS completely blocked the sphere reformation ability in both cell lines (Figure 4g,h). Hypoxia induced significantly higher clonogenic activity in both cell lines which could not be reversed by the clinically used chemotherapeutic drugs (Figure 4i,k). In contrast, the sphere reformation and hypoxia-induced clonogenicity were completely blocked by DS/Cu (Figure 4g–k). In line with our previous reports [26,27], the effect of DS on NSCLC sphere reformation and clonogenicity is copper-dependent. In combination with copper, DS reverses hypoxia-induced chemoresistance to four tested drugs (Figure 4l). Isobologram indicates a synergistic effect between DS/Cu and the anticancer drugs (Table 3). These results are highly consistent with our previous studies in other types of cancer [8,16,17,18,22,23,27,48,49].

Although DS shows strong cytotoxicity *in vitro*, the clinical application of DS in cancer therapeutics has been limited by its very short half-life in the bloodstream [23,50]. To overcome this bottleneck, our team developed a PLGA-nanoparticle encapsulated DS to protect DS from degradation in the bloodstream which shows very optimistic efficacy in mouse GBM and liver cancer models [8,23]. In this study, we examined the efficacy of DS-PLGA in a metastatic NSCLC mouse model. After intravenous injection of A549 cells, multiple cancer nodules were observed in the lungs of the control mice, especially on the edge of the lung (Figure 5a). The micrographic images show solid infiltration of cancer cells in the lungs of the control mice (Figure 5c,d). This observation was further supported by the heavier lung weight of the control mice (Figure 5b). In comparison with the control group, very few cancer nodules were observed in the lungs of the DS-PLGA/Cu treated mice (Figure 5a,c,d). In line with our previous studies [8,23], all the mice in the treated group tolerated the treatment very well and no body weight loss was observed.

## 5. Conclusions

Our findings revealed that hypoxia is the key regulator of the stemness in NSCLC cells. The hypoxia-induced NSCLC CSCs are highly resistant to the clinically used anti-NSCLC chemotherapy drugs. Disulfiram, an anti-alcoholism medicine, demonstrated strong anti-CSC activity and reversed hypoxia-induced chemoresistance. The promising *in vivo* anticancer efficacy indicates that further investigation might translate DS-PLGA into NSCLC clinical application.

## Figures and Tables

**Table 1:** IC_50_ values in A549 and NCI-H23 cells treated with drugs.

	A549		H23	
Drug	Normoxia	Hypoxia	Normoxia	Hypoxia
OXA (µM)	1.4 ± 0.3	>25	9.0 ± 5.2	>25
DOX (nM)	56.6 ± 9.3	>1000	292.5 ± 8.2	>1000
dFdC (nM)	12.7 ± 2.7	>100	9.6 ± 3.2	>100
PTX (nM)	4.2 ± 3.4	>100	8.7 ± 6.1	>100
DS/Cu nM	1.0 ± 0.3	5.9 ± 3.0	13.8 ± 2.3	8.7 ± 1.0

**Table 2:** The IC_50_ anti-NSCLC drugs in the transfected clones.

Drug	Mock	NFκB-p65	
		C7	C13
OXA (µM)	6.7 ± 1.5	>25	>25
DOX (nM)	453.6 ± 240.3	585.2 ± 241.9	706.7 ± 287.9
dFdC (nM)	24.9 ± 20.4	>100	>100
PTX (nM)	2.4 ± 0.5	23.5 ± 23.3	37.2 ± 22.2

**Table 3:** Synergistic effect of chemotherapy drugs and DS/Cu on NSCLC cell lines.

Drug	A549 Hypoxia					H23 Hypoxia				
	*IC_50_*		CI Values			*IC_50_*		CI Values		
	*Sing*	*Com*	ED50	ED75	ED90	*Sing*	*Com*	ED50	ED75	ED90
DOX (nM)	>100	*3.4 ± 2.1*	0.92	0.86	0.81	>100	*10.6 ± 5.2*	0.81	0.79	0.77
dFdC (nM)	59.5* ± 29.1*	*1.8 ± 0.5*	0.66	0.34	0.18	>100	*7.0 ± 2.1*	0.64	0.52	0.48
OXA (µM)	>10	*1.2 ± 11.1*	0.93	0.98	1.02	>10	*0.8 ± 0.3*	0.84	0.83	0.83
PTX (nM)	8.9* ± 3.0*	*4.5 ± 0.7*	0.95	0.69	0.78	>100	*6.7 ± 1.5*	0.80	0.63	0.58

Sing: Single drug; Com: Drug in combination with DS/Cu.

## Data Availability

Data are available upon request.

## Supplementary Materials

The following are available online at https://www.mdpi.com/article/10.3390/biom14121651/s1.
