# Supplementary figures and images for: PLGA-Nano-Encapsulated Disulfiram Inhibits Cancer Stem Cells and Targets Non-Small Cell Lung Cancer In Vitro and In Vivo
Source: Biomolecules. 2024 Dec 23;14(12):1651. doi: 10.3390/biom14121651 (PMC11674892; doi:10.3390/biom14121651)

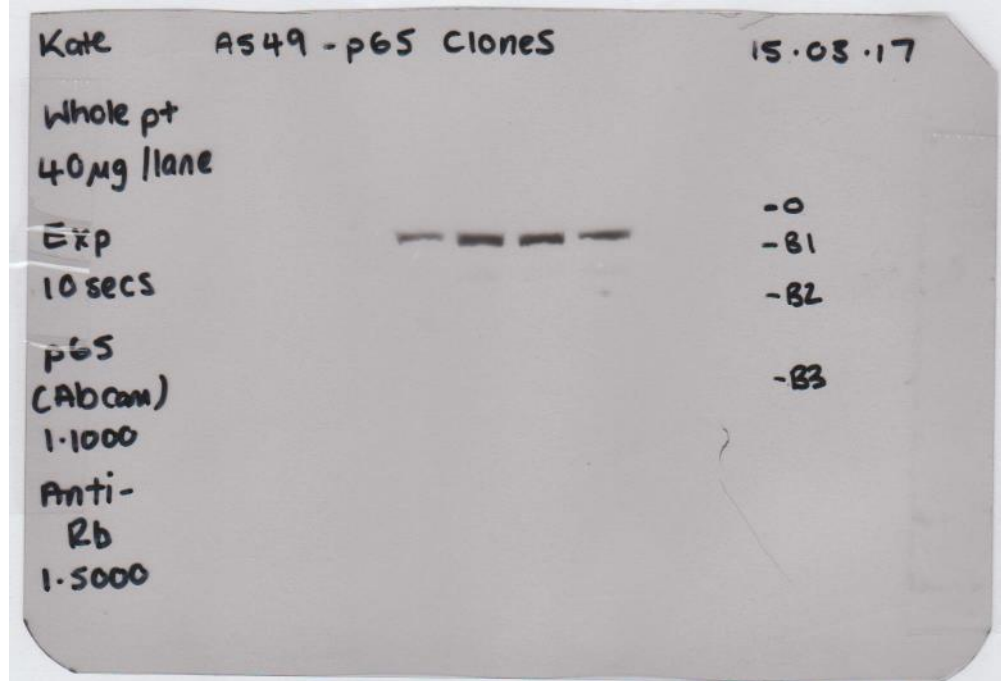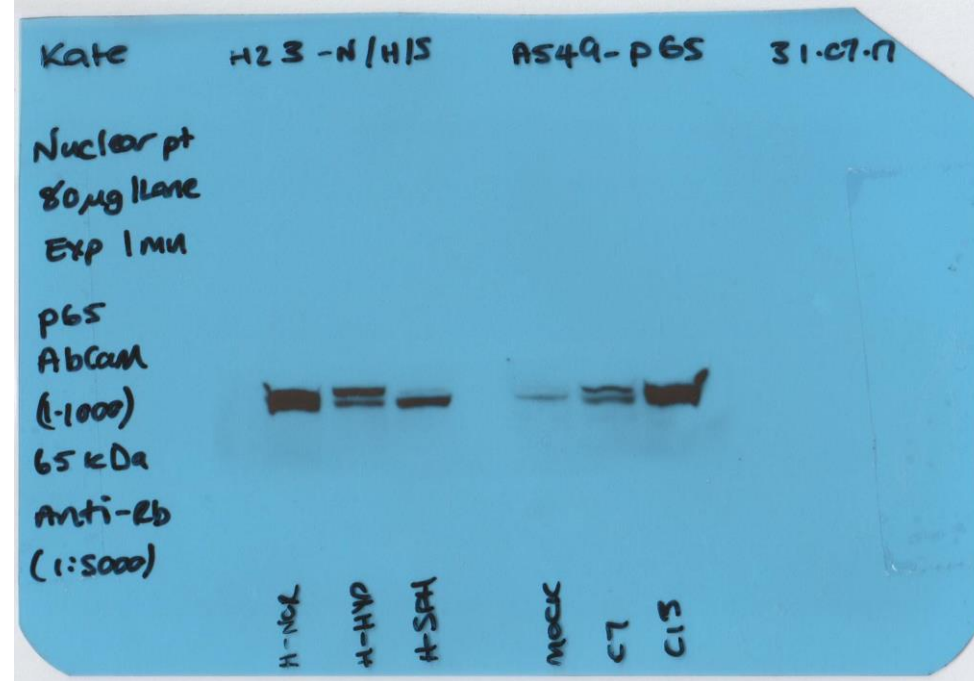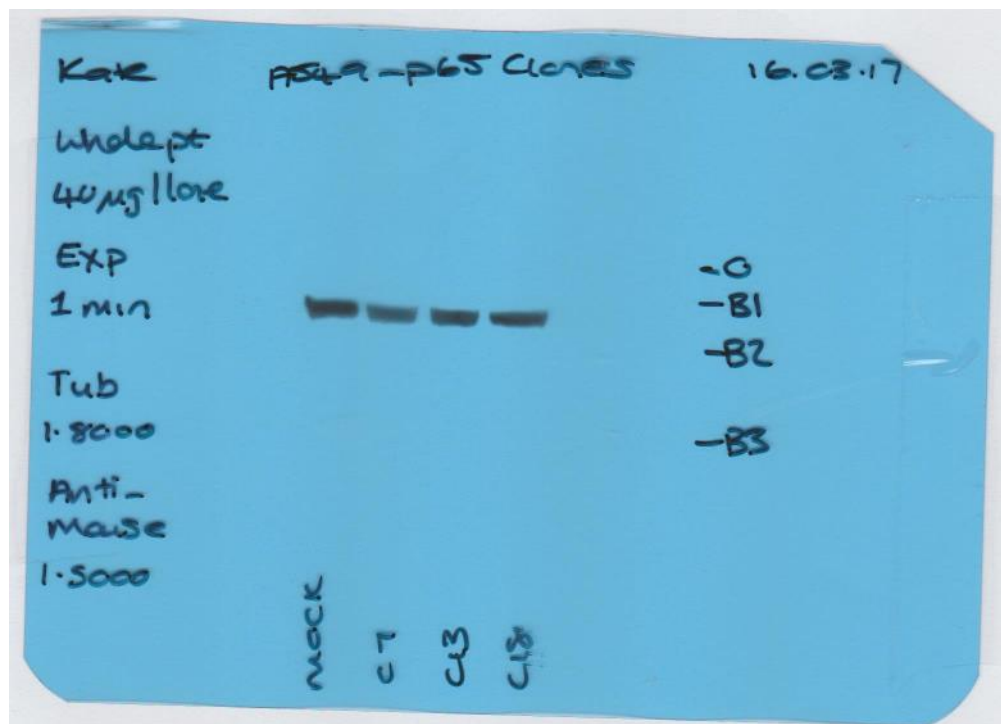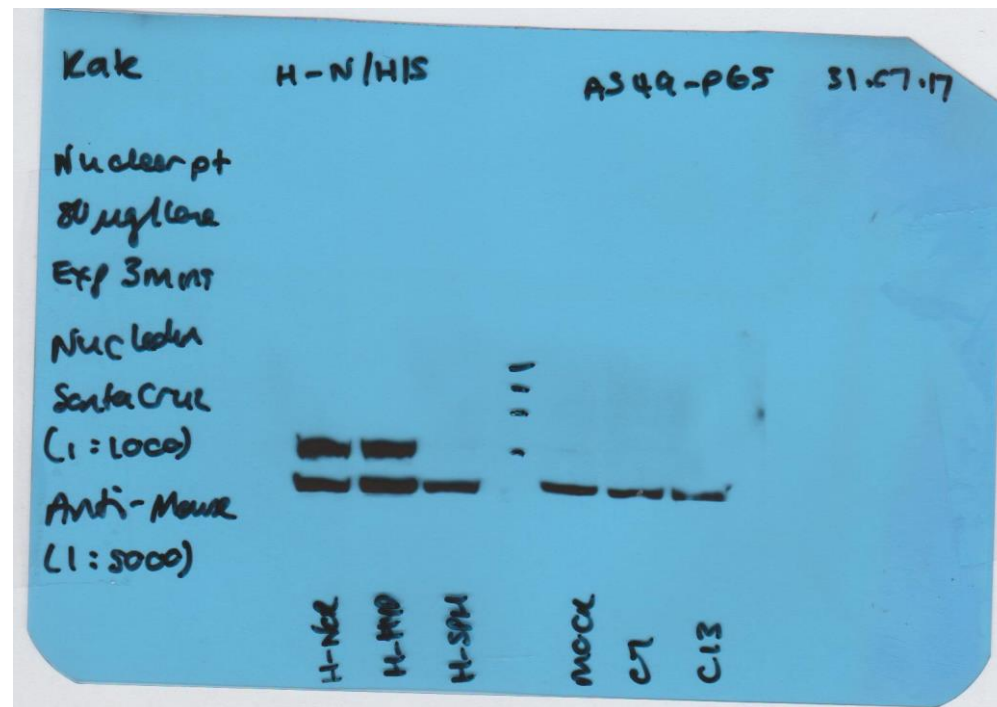

Supplement: Supplementary file 1 [file biomolecules-14-01651-s001.zip › biomolecules-3299881-supplementary.pdf]
